# Universal Behaviors as Candidate Traditions in Wild Spider Monkeys

**DOI:** 10.1371/journal.pone.0024400

**Published:** 2011-09-19

**Authors:** Claire J. Santorelli, Colleen M. Schaffner, Filippo Aureli

**Affiliations:** 1 Department of Psychology, University of Chester, Chester, United Kingdom; 2 Research Centre in Evolutionary Anthropology and Palaeoecology, School of Natural Sciences and Psychology, Liverpool John Moores University, Liverpool, United Kingdom; Yale University, United States of America

## Abstract

Candidate traditions were documented across three communities of wild spider monkeys (*Ateles geoffroyi*) using an *a priori* approach to identify behavioral variants and a statistical approach to examine differences in their proportional use. This methodology differs from previous studies of animal traditions, which used retrospective data and relied on the ‘exclusion method’ to identify candidate traditions. Our *a priori* approach increased the likelihood that behavior variants with equivalent functions were considered and our statistical approach enabled the proportional use of ‘universal’ behaviors, i.e., used across all communities, to be examined for the first time in any animal species as candidate traditions. Among universal behaviors we found 14 ‘community preferred’ variants. After considering the extent to which community preferred variants were due to ecological and, to a lesser degree, genetic differences, we concluded that at least six were likely maintained through social learning. Our findings have two main implications: (i) tradition repertoires could be larger than assumed from previous studies using the exclusion method; (ii) the relative use of universal behavior variants can reinforce community membership.

## Introduction

Observational studies of inter-population behavioral variation have identified a growing number of species that have traditions (reviewed in [1]–[3]). Such studies are crucial for understanding the role and function of traditions in the context of environmental selection pressures faced by individuals [4]. Commonly, evidence for traditions is acquired using the ‘exclusion method’ that requires the habitual presence of a behavior in at least one community and its absence in another community, which cannot be accounted for by ecological or genetic differences alone [5], [6]. ‘Universal’ behaviors, i.e., those present across all communities, are usually neglected when searching for convincing evidence for traditions. Therefore, inter-community variation in the proportional use of universal behaviors may have gone unreported in previous studies. However, universal behaviors might still be the result of innovation and transmission by social learning within multiple geographically distinct communities [6].

Much behavioral variation is thought to be adaptive, giving individuals a selective advantage (e.g., a foraging technique that enables access to a nutritious food resource previously denied [7], [8]). However, the function of some behavior variants, especially in the social domain, may not be as obvious [9], [10]. Of particular interest are behavioral variations that cannot be explained through ecological or genetic factors alone, yet persist despite any obvious adaptive value [11]. Socially learned behaviors that show no obvious adaptive function in free-ranging populations include stone-handling by Japanese macaques (*Macaca fuscata*) [12], tool-use for drinking water in immature chimpanzees (*Pan troglodytes)* [13], chimpanzee grooming postures [14], [15], and differences in burrow emergence times in meerkats (*Suricata suricatta*) [16]. However, it is possible that many socially learned behaviors without an obvious adaptive value have gone unnoticed in studies on wild populations and their variation across communities not explored [17].

Furthermore, individuals may be exposed to multiple variants of a behavior, which offer functionally equivalent alternatives, where differences in available alternatives have no proximate consequences. Captive studies focusing on social learning or transmission mechanisms involve experiments with functionally equivalent ‘two-action’ tasks for just this reason (see [18]). Although for functionally equivalent behaviors there is no inherent advantage to performing one version over another, over time a tendency to behave similarly to those nearby and subsequently selection for conformity-enforcing behaviors might emerge [19], [20].

Many human cultural traits function as identity-signaling behaviors (e.g., attitudes, possessions, and rituals) in order to avoid the costs of misidentification [21]. Therefore, it is possible that some animal traditions, which offer no functional advantage over alternate variants, emerge and persist as identity-signaling behaviors, which becomes an additional function. Accurate signals of group membership, for example, may be particularly important for species in which groups are relatively fluid and members may not be in contact for prolonged periods, necessitating frequent and rapid recognition of familiar individuals [22].

Spider monkeys (*Ateles* spp.) possess key characteristics that provide favorable opportunities for social learning [23]–[25], including long infant dependence, a long life span [26] and social tolerance [27]. Additionally, spider monkeys live in communities with a high degree of fission-fusion dynamics, in which members are rarely all together and split and merge into subgroups of variable membership [27], [28]. Under these conditions recognition of community membership is important, and variation in behaviors that are functionally equivalent could potentially supply signals of group identity.

We have already documented the presence of 22 traditions across five populations of spider monkeys using the exclusion method [29]. The behaviors analyzed in the previous study were selected retrospectively and therefore incorporated behaviors that were collected by observers as part of datasets that focused on other research topics. In the present study, our aim was to document traditions in spider monkeys using an innovative approach. Firstly, we used an *a priori* approach based on detailed descriptions of behaviors in order to capture the relative occurrence of potential variants in our three study populations, and this resulted in a dataset that was almost completely independent from our previous study [29]. Secondly, instead of focusing on variation across communities in categorical terms of absence and extent of presence (e.g. habitual, customary) of behavioral traits, we examined inter-community variation across and within sites by statistically analyzing the relative occurrence of universal behaviors. As in Kendal et al. [17] our approach relies on the accepted assumption within social learning research that greater intra-group homogeneity of behavioral variants emerges in the presence of social learning than would be expected without social learning, once genetic and ecological differences are accounted for. We predicted that even among universal behaviors, evidence for ‘community preferred’ variants would be established. After considering the extent to which inter-community variation was due to social learning rather than ecological and, to a lesser degree, genetic differences across sites, we suggest which community preferred variants could be considered candidate traditions.

## Results and Discussion

Behavioral data were collected from three communities of spider monkeys (*Ateles geoffroyi*): two neighboring communities at Punta Laguna, Mexico (hereafter PL-East and PL-West), and one at Santa Rosa, Costa Rica (hereafter Santa Rosa) (Table 1). We tested for differences in the proportional use of behavior variants across communities. Out of 36 behaviors with sufficient sample size for statistical analysis (Table S1), their proportions were not significantly different among communities in 22 cases (Table S2). Proportions differed significantly among communities for the 14 remaining behaviors (Table 2) and are reported with their significant post-hoc test *P* values below.

**Table 1:** Composition of the three study groups.

	2006			2007		
	Male	Female	Total	Male	Female	Total
**Santa Rosa** *						
Adult	6	9	15	6	8	14
Sub-adult	2	7	9	2	6	8
Juvenile	0	0	0	0	0	0
Infant	4	2	6	3	2	5
Total	12	18	30	11	16	27
**Punta Laguna – East**						
Adult	2	8	10	1	8	9
Sub-adult	1	0	1	3	3	6
Juvenile	2	2	4	2	2	4
Infant	5	2	7	4	0	4
Total	10	12	22	10	13	23
**Punta Laguna – West**						
Adult	5	9	14	5	8	13
Sub-adult	1	2	3	1	2	3
Juvenile	3	4	7	3	4	7
Infant	2	1	3	2	2	6∧
Total	11	16	27	11	16	29

Adult = older than 8 years; sub-adult = 5–8 years; juveniles = 3–5 years; infants 0–3 years; an individual younger than 3 years but whose mother had already another offspring were considered juveniles [54].

*Demographic data for age class classification were not available for older individuals and so individuals were classified based on size. Sub-adults were individuals that moved independently from their mother (i.e. could be found in subgroups where the mother was not present) and were sexually mature, but were not fully adult size.

∧ Includes two individuals of unknown gender.

**Table 2:** ANOVA results for behaviors that varied significantly across communities.

Domain	Sub-domain	Behavioral variant	*F*	df	*P*	Mean proportion ± SE		
						Santa Rosa	PL-East	PL-West
Fruit extraction method	Tail-assisted suspension	hand instead of mouth	6.058	2,41	0.005	0.422±0.054	0.145±0.059	0.237±0.069
	Tail-assisted standing	hand instead of mouth‡ ф	5.753	2,31	0.008	0.260±0.054	0.057±0.054	0.375±0.105
Drinking	Drink style	lick instead of dribble‡	5.583	2,28	<0.001	1.000±0.063	0.741±0.59	0.970±0.071
	Hand use	left hand instead of right hand‡ ф	3.133	2,44	0.053	0.625±0.095	0.485±0.087	0.848±0.110
Ground use	Ground use	foraging	4.190	2,23	0.028	0.111±0.107	0.506±0.076	0.833±0.132
Marking	Substrate marking	chest rub†	44.468	2,58	<0.001	0.558±0.038	0.019±0.065	0.000±0.000
		genital rub†	38.650	2,58	<0.001	0.134±0.047	0.865±0.080	0.773±0.087
Greetings	Approach type	approach with a greeting instead of without a greeting	6.205	2,90	0.003	0.091±0.016	0.050±0.021	0.047±0.019
	Greeting type	contact greeting instead of non-contact greeting‡	13.443	2,51	<0.001	0.671±0.047	0.598±0.091	0.071±0.109
	Pectoral sniff use	pectoral sniff with embrace instead of without embrace*			0.017	0.798±0.047	0.573±0.073	n/a
Resting	Association when close	resting in proximity†	5.803	2,25	0.009	0.461±0.039	0.394±0.046	0.205±0.073
	Substrate size	medium size†	7.523	2,40	0.002	0.406±0.036	0.270±0.040	0.197±0.049
	Resting posture	sitting upright† ‡ ф	14.402	2,40	<0.001	0.373±0.039	0.657±0.042	0.642±0.052
		leaning lateral† ‡ ф	11.872	2,40	<0.001	0.479±0.037	0.232±0.04	0.246±0.049

PL-East = Punta Laguna - East community; PL-West = Punta Laguna - West community;

*As the Punta Laguna - West community could not be included in the analysis an independent t-test was performed (see text);

† Significant after applying Bonferroni's correction (see Methods);

‡ identified as a community preferred behavior likely maintained by social learning;

ф functionally equivalent variant.

### Fruit extraction methods

While feeding in a tail assisted suspension posture, individuals in Santa Rosa used their hands, instead of their mouths, to extract fruit proportionally more than individuals in PL-East (*P* = 0.007) and PL-West (*P* = 0.039). In addition, during tail-assisted standing, individuals in PL-East used their hands, instead of their mouths, less than individuals in Santa Rosa (*P* = 0.015) and PL-West (*P* = 0.046).

If visual cues alone were sufficient for evaluating fruit ripeness, hand extraction might be a more efficient method of consumption. However, the lack of variation in extraction methods during sitting posture (Table S2) suggests that determining fruit ripeness is not the main function of extraction behaviors. The significant difference in the use of hand relative to mouth extraction between the neighboring Punta Laguna communities, which are unlikely to differ in the availability of fruit size or ripeness, suggests that this variation is not due to ecological differences. In a food retrieval task, male spider monkeys (*Ateles geoffroyi*) were reported to retrieve food using their mouth instead of their hand significantly more often than females [30]. As the proportion of males in PL-East was not higher than in the other two communities (Table 1), the possible male preference could not account for the relative bias towards mouth extraction observed in this community. The relative preference of hand extraction over mouth extraction for PL-West and Santa Rosa individuals in contrast to PL–East individuals may therefore be a community preferred behavioral variant possibly maintained by social learning.

### Drinking

Of the two drinking variants used to obtain water from ground sources or tree holes, licking was more common than dribbling among all individuals of the three communities (Table 2). Licking was the only drinking variant used by Santa Rosa individuals, and was used exclusively by all but one individual in PL-West. However, PL-East individuals used the licking style less often than individuals in Santa Rosa (*P* = 0.01), and PL-West (*P* = 0.044). It is difficult to know whether one drinking variant was more efficient than the other. However, as almost all PL-West individuals only performed the licking variant and neighboring PL-East individuals performed both variants (6 individuals used both variants, 7 individuals only licked, 1 individual only dribbled) ecological differences cannot account for this within-site variation.

Similarly, there was variation in hand use when drinking between neighboring Punta Laguna communities, with individuals in PL-West using their left hand instead of their right hand more often than individuals in PL-East (*P* = 0.042). A meta-analysis found that most primate individuals are lateralized for practiced tasks [31]. Whereas no studies have investigated handedness in water-related activities in spider monkeys, there is evidence of a right-hand preference for this behavior in captive chimpanzees [32], [33]. While 59% of SR individuals and 58% of PL-East individuals exclusively used one hand (either their left or right) to drink water, 87.5% of PL-West individuals did so, 75% of which exclusively used their left hand. This unexpected result from the PL-West community does correspond with research showing a left hand bias at the individual and group level for food-reaching tasks in thirteen captive spider monkeys [30]. Although our sample size was small, and other factors (e.g. the importance of tactile cues in haptic tasks [33]) may play a role, the contrast between the two Punta Laguna communities suggests that hand use when drinking is influenced by social learning.

### Ground use

Individuals in Santa Rosa used the ground for foraging relatively more than PL-East (*P* = 0.04) and PL-West (*P* = 0.027) individuals. Spider monkeys are rarely on the ground [34] and predation on them by jaguars (*Panthera onca*) and pumas (*Puma concolor*) occurs at both sites (Punta Laguna: G. Ramos-Fernandez, personal communication; Santa Rosa: G. McCabe, personal communication). It is possible however that subtle inter-site differences, such as continuity of canopy cover, predation risk and the availability of preferred ground foods, might differentially affect the advantages and risks associated with ground use. Therefore, caution should be exercised when considering ground use for foraging as a potential tradition.

### Marking

Chest rubbing was the most common form of marking behavior at Santa Rosa and occurred proportionally more than in the Punta Laguna communities (*P*<0.001 in both cases) as it was observed only once among PL-East individuals and never among PL-West individuals (Table 2). In contrast, genital rubbing was the most common form of marking behavior in both Punta Laguna communities and occurred relatively more than at Santa Rosa (*P*<0.001 in both cases).

Sternal and genital glands convey distinct information in many mammals [35], therefore it is likely that the olfactory message conveyed by chest rubbing is different from that of genital rubbing (e.g. territorial versus reproductive respectively), and their use may differ between males and females [36], [37]. Thus, the inter-site variation in marking may arise because of the differential occurrence of stimuli at the two sites (e.g. more territorial activities at Santa Rosa). However, data were not collected on either marking location or individuals' reproductive status to test such a hypothesis. Thus, although the extreme rarity of chest rubbing at Punta Laguna remains intriguing and the role of social learning cannot be ruled out, ambiguity regarding the function of the two marking variants excludes them from being considered as likely traditions at this stage.

### Greetings

Although the majority of approaches in all communities were not followed by a greeting (Table 2), individuals in the Santa Rosa community followed an approach with a greeting more often than individuals in PL-West (*P* = 0.003). Furthermore, Santa Rosa and PL-East individuals gave more contact greetings relative to non-contact greetings, than PL-West individuals (*P*<0.001 and *P* = 0.001, respectively). In addition, Santa Rosa individuals gave more pectoral sniffs in combination with an embrace than PL-East individuals (independent *t* test: *t*_35_ = 2.150, *P* = 0.017; as only one pectoral sniff was observed in PL-West, this community was excluded from the analysis).

Spider monkeys regulate their social relationships using embraces and pectoral sniffs to signal benign intent [38]. For example, the rate of embraces a female receives increases when she has a young infant [39]. The numbers of infants present in Santa Rosa and Punta Laguna communities were roughly the same (10 in Santa Rosa; 9 in PL-East and 13 in PL-West) suggesting differences in infant numbers are unlikely to explain inter-community variation for greeting behavior. However, hourly approach rates differed across communities (ANOVA: *F*_2,46_ = 17.744, *P* = <0.001), with Santa Rosa individuals approaching others (mean ±SE = 3.26±0.307, *N* = 19) more often than PL-East individuals (mean ±SE = 1.051±0.335, *N* = 16) and PL-West individuals (mean ±SE = 0.792±0.358, *N* = 14). This difference may suggest that Santa Rosa individuals experienced more uncertainty when community members approached, which necessitated signaling benign intentions more often. Furthermore, the risk associated with approaches could be lower for Punta Laguna individuals, thus signals to mitigate threats were used less frequently.

The risks associated with non-contact greetings are lower than with contact greetings, which involve close body contact and expose vulnerable body parts to harm [40]. It is difficult to determine whether contact versus non-contact greetings are influenced by the regulation of social interactions, which can be prone to rapid change [27]. However, social learning is likely to play a role in maintaining these community preferred behaviors regardless of whether the need for such regulation differs across communities, because of the substantial difference in the use of the two greeting variants between the Punta Laguna communities.

### Resting

Of the three variants of degrees of physical closeness individuals used when resting together, only the use of resting in proximity varied significantly across communities (Table 2). Individuals in PL-West used resting in proximity significantly less often than in Santa Rosa (*P* = 0.006) and PL-East (*P* = 0.046). Predation risk is likely similar across communities (see Ground use), and ambient temperature does not differ between the Punta Laguna communities. Accordingly, social factors may be critical to explain why PL-West individuals were more likely to stay in close physical contact, rather than in proximity, when resting near one another. For example, resting associations may reflect different levels of affiliation, and PL-West individuals may signal strong affiliative bonds through resting in physical contact. In the other communities such bonding displays may not be required, or may be given using other behaviors. This ambiguity surrounding whether resting association variants have discrete functions or are equivalent, makes it difficult to determine whether their expression is due to a learned community preferred resting association, or to differences in community social dynamics.

Of the three branch sizes of resting substrate examined, the proportion of medium sized substrates varied significantly across communities, with Santa Rosa individuals using them relatively more often than PL-West individuals (*P* = 0.001). Ecological differences impacting on variation in substrate use between sites might include size or weight differences across focal animals, or differences in substrate availability. Although no data are available for either factor, there are no obvious consistent physical differences in individual body appearances between the two sites. It is possible that hurricane damage sustained across the Yucatan region in 2005 temporarily reduced the availability of larger sized branches at the Punta Laguna field site [41] and thus an ecological explanation cannot be ruled out.

Resting in an upright sitting position was the most popular posture among Punta Laguna individuals who used it relatively more often than Santa Rosa individuals (PL-East: *P*<0.001; PL-West: *P* = 0.001). In contrast, the leaning lateral posture was relatively more common among Santa Rosa individuals than among PL-East (*P*<0.001) and PL-West individuals (*P* = 0.001). Individuals of the three communities regularly performed all four variants of resting posture providing individuals with frequent social learning opportunities for each variant. Therefore, the existence of community preferred resting postures, despite knowledge and use of other variants, suggests a social learning component could be involved in maintaining this community variation.

In order to address the potential bias of resting postures resulting from possible differences in substrate availability across sites, an analysis on how each substrate size was used in conjunction with each of the four resting postures was conducted. There was no significant difference in resting postures for small or large substrates across the three communities. However, for medium sized substrates there were differences in the proportion of leaning lateral (*P*<0.001) and sitting postures used (*P* = 0.006), which correspond to the findings for overall resting postures across communities (see above). Thus, potential inter-site difference in availability of substrate size cannot be the explanation for variation in resting postures, as the use of ‘community preferred’ resting postures was only significantly used by individuals in medium sized branches.

### Conclusions

The *a priori* approach used in this study enabled an examination of behavior variants that may have been overlooked in previous tradition studies, which used retrospective data originally collected for other purposes. The statistical analysis of the proportional use of universal behaviors revealed 14 differences across communities. These results support our prediction that even among universal behaviors, evidence for community preferred variants can be established. We then evaluated whether any of these 14 differences could simply be explained by ecological variation across the sites (see Results and Discussion). This was not the case for at least six universal behavior variants (i.e., hand instead of mouth fruit extraction when tail assisted standing, drinking by licking instead of dribbling, drinking with the left hand instead of the right hand, contact instead of non-contact greeting, resting in an upright sitting posture and resting in a leaning lateral posture; Table 2), which were likely maintained through social learning and would not have been considered as candidate traditions using the exclusion method [6]. This is a conservative estimate of candidate traditions as we did not include cases in which socio-ecological explanations could not be ruled out. In addition, four of these six candidate traditions (i.e. hand instead of mouth to extract fruit, drinking with left hand instead of right hand, resting in an upright sitting posture and resting in a leaning lateral posture) were functionally equivalent behaviors because no differential advantage in the use of one variant over another across communities was evident. In contrast, the variants of the other two candidate traditions (i.e. drinking by licking instead of dribbling and contact instead of non-contact greeting) potentially differ in their function or energetic efficiency. For example, the energetic costs associated with dribbling water into the mouth may be different than those for licking water off the fist, and different greeting types likely reflect differences in tolerance between individuals.

Genetic differences between populations at the two sites cannot be ruled out as an explanation of inter-site differences [42]–[44]. However, variation in community preferred behaviors was not restricted between sites, but also occurred between the two communities at the same site, which belong to the same population. In the cases in which an ecological explanation was ruled out (see Results and Discussion), this lack of restriction suggests that social learning was more influential in determining inter-community behavioral variation than genetic differences.

Our findings have two main implications. Firstly, the tradition repertoire of other species could be larger than assumed from previous studies. The exclusion method is a highly successful tool in documenting traditions [5], [25], [29], [45]–[47], but it does not allow consideration of universal behaviors. Complementing the exclusion method with statistical analysis of the relative use of behavioral variants across groups would allow the inclusion of universal behaviors and a more complete assessment of the tradition repertoire of any species (cf. [6], [17]).

Secondly, the relative use of behavioral variants could contribute to the recognition of group membership if one potential role of behavioral variants, which offer no obvious direct advantage over alternate variants or are functionally equivalent, is to serve as identity-signaling behaviors [note that as socially learned behavioral variants tend towards homogeneity within a population even if they do not function as identity signaling behaviors [17], it should not be presumed that the putative function of such behaviors is to act only as identity-signaling behaviors]. Whereas the presence of one or multiple behavioral variants can be used as signals of group membership when individuals meet after prolonged separation [22], conformity to the relative use of behavioral variants may reinforce group membership [21]. Both aspects are particularly important in species, like spider monkeys, living in communities with a high degree of fission-fusion dynamics. The social fluidity can challenge the maintenance of community identity. This could be compensated by an enhanced propensity to conform to the relative use of behavioral variants of other community members. This propensity could be particularly relevant for immigrant spider monkeys as moving into a new community is risky [48] and may overall steer social learning opportunities (e.g., attending to long-term residents rather than newly immigrant individuals [49]; or discriminating group members based on their tool-using skills [50]). Thus, not only unique traditions, but also the relative use of universal behaviors can play an important role in identifying community membership.

## Methods

### Ethics Statement

This study was carried out in the field with free-ranging monkeys and was completely observational. Research was conducted at all times in accordance with the laws of participating countries. Permission to conduct research was granted by the University of Chester Psychology Department Ethics Committee and approved by the University of Chester Animal Ethics Committee, the Costa Rica Ministry of Environment and Energy (MINAE) permit #ACG-PL-030-2006 and the Mexican government under the auspices of Pronatura, Peninsula de Yucatan, A.C. (PPY) #1577105.

### Study sites and subjects

Data were collected from three communities of wild spider monkeys (*Ateles geoffroyi*; Figure S1) who were individually identifiable using unique facial and body characteristics. Two neighboring communities ranged within the Otoch Ma'ax Yetel Kooh reserve, Punta Laguna, Yucatan Peninsula, Mexico. The third community ranged within Santa Rosa National Park, Area de Conservacion Guanacaste, Costa Rica. These two distinct geographic regions are ecologically similar, tropical dry forests with a severe dry season between December and May [51], [52]. The Santa Rosa community consisted of 27–30 individuals, the PL-East community of 22–23 individuals and the PL-West community of 27–29 individuals (Table 1).

### Data collection

Behavioral data were collected over 18 months during 2006 and 2007 using previously defined categories (i.e., the *a priori* approach). Each year data were collected for 4.5 months at each site, such that each site was monitored during a wet and a dry season. Data were collected using 15-minute focal observations on all adult, sub-adult and juvenile individuals with continuous and instantaneous sampling every 30 seconds. Additional observations of rare behaviors were collected on an *ad libitum* basis. An attempt was made to record similar numbers of focal observations across individuals and communities. However, due to the high degree of fission-fusion dynamics this was not always possible.

### Data analysis

All behaviors were categorized into domains and sub-domains. Only the 36 behaviors with a sufficiently large sample size were included in statistical analysis (Table S1; Figure S2). For each individual, the proportion of performance of each behavioral variant was calculated out of the corresponding sub-domain total. For one domain, additional data from a pilot study conducted in 2004–05 were included to provide a larger sample size for the sub-domain totals and calculate proportions more accurately (Table S1). To ensure a reliable proportion, individuals who had less than two hours of focal observations were excluded from analyses (overall mean ±SE focal duration: 440.1±39.3 minutes; Santa Rosa = 599.5±80.2 minutes; PL-East = 414.4±23.5 minutes; PL-West = 233.3±24.2 minutes). Individual proportions were transformed using the arcsine square-root to normalize the data [53] before using them to test for differences across communities with one-way analysis of variance (ANOVA) or independent t-tests. The post-hoc test Tukey's HSD was applied following significant ANOVA results. All tests were two-tailed with alpha levels set to 0.05, and Bonferroni's correction was applied when multiple tests within a sub-domain were conducted. All tests were conducted in SPSS 15.0.

## Supporting Information

Figure S1 **Central America map showing location of study sites.** Arrows illustrate location of participating field sites within their host country.
(TIF)

Figure S2 **Photographs of some behavior variants examined** (Photo credit Claire J. Santorelli unless otherwise stated).
(TIF)

Table S1 Behavioral variants with a sufficiently large sample size for statistical analysis and their domains and sub-domains. ^†^Additional data collected between 2004 and 2005 was also used for analysis.
(TIF)

Table S2 ANOVA results for the behavioral variants that did not differ significantly across communities. PL-East = Punta Laguna – East community; PL-West = Punta Laguna – West community; *Not significant as critical value is 0.013 when Bonferroni's correction was applied (see Methods); ^†^Not significant as critical value is 0.017 when Bonferroni's correction was applied (see Methods).
(TIF)
