# Supplementary figures and images for: Universal Behaviors as Candidate Traditions in Wild Spider Monkeys
Source: PLoS One. 2011 Sep 19;6(9):e24400. doi: 10.1371/journal.pone.0024400 (PMC3176216; doi:10.1371/journal.pone.0024400)

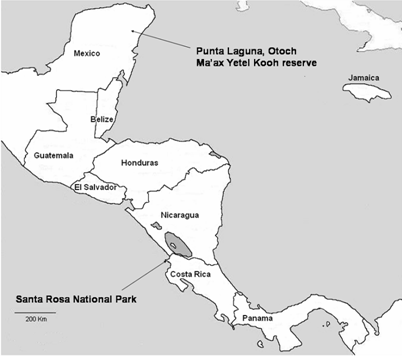

Supplement: Figure S1 — Central America map showing location of study sites. Arrows illustrate location of participating field sites within their host country. (TIF) [file pone.0024400.s001.tif]

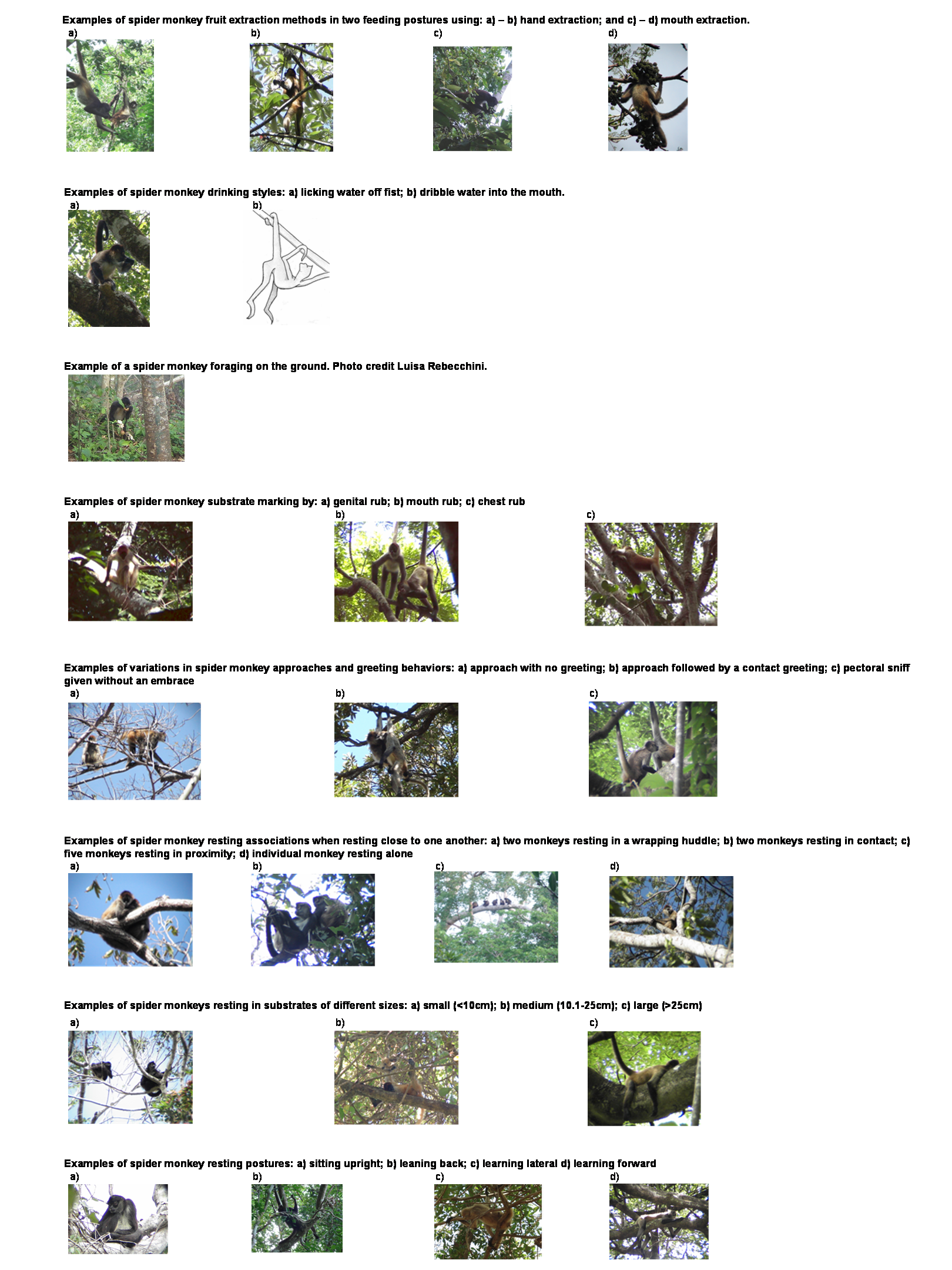

Supplement: Figure S2 — Photographs of some behavior variants examined (Photo credit Claire J. Santorelli unless otherwise stated). (TIF) [file pone.0024400.s002.tif]

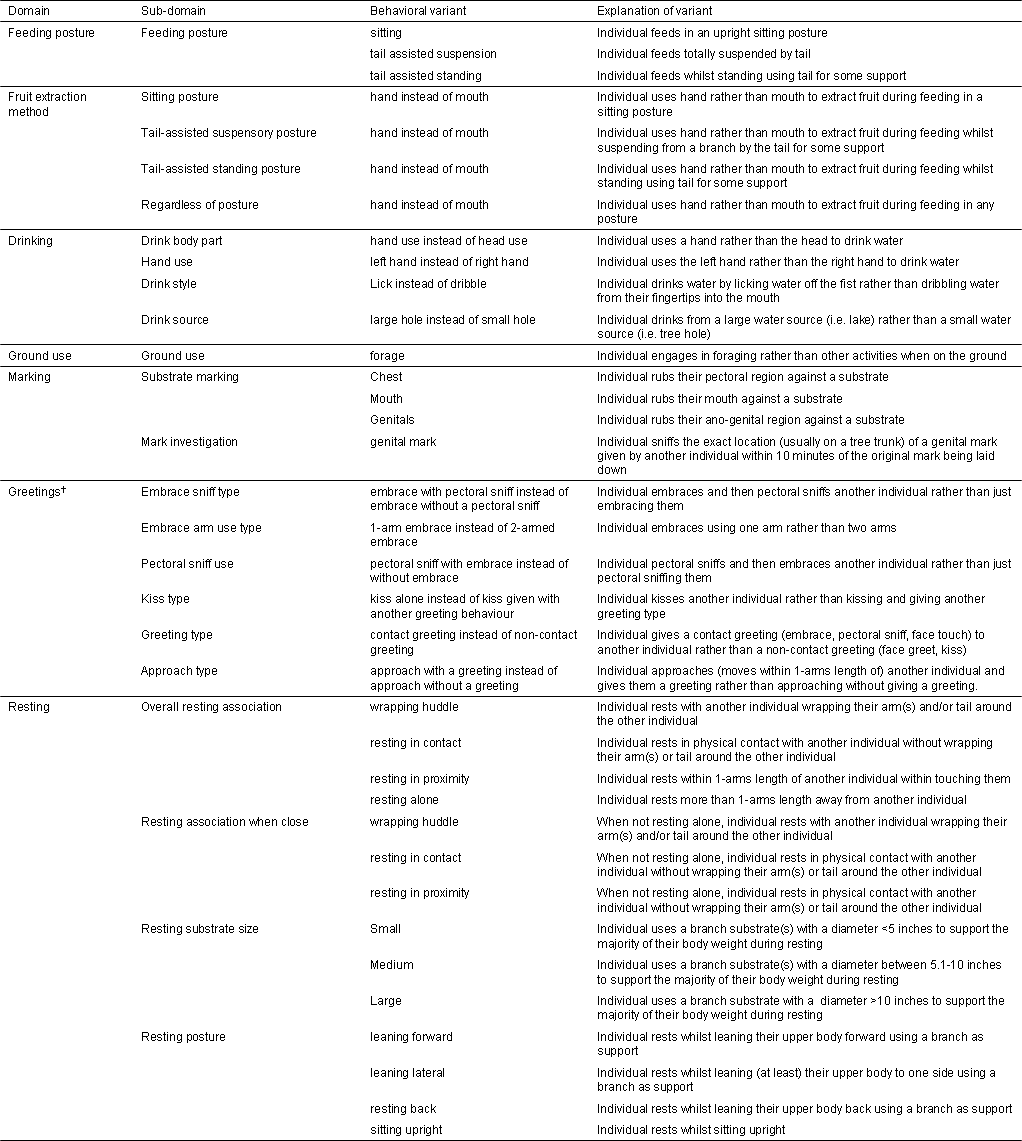

Supplement: Table S1 — Behavioral variants with a sufficiently large sample size for statistical analysis and their domains and sub-domains. †Additional data collected between 2004 and 2005 was also used for analysis. (TIF) [file pone.0024400.s003.tif]

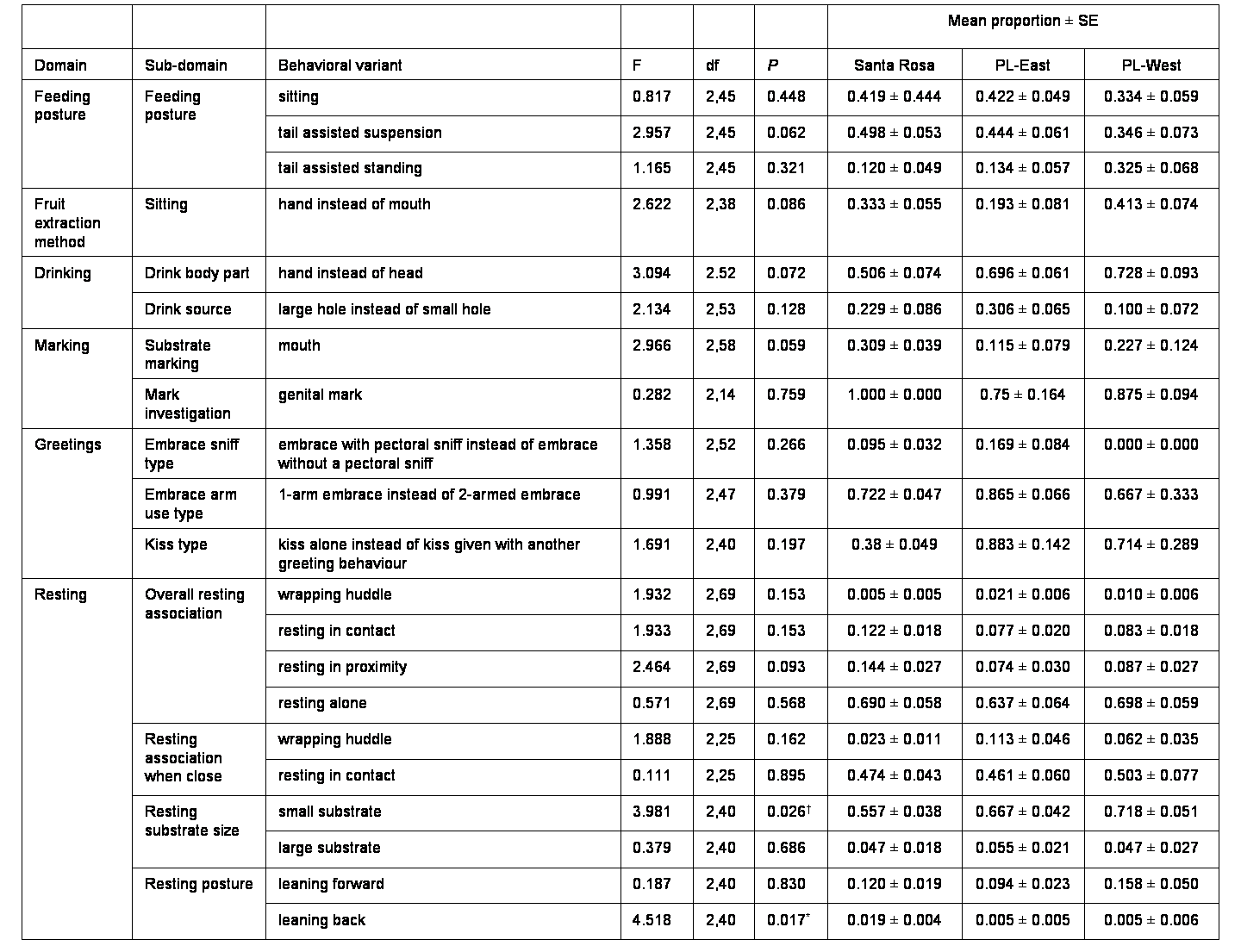

Supplement: Table S2 — ANOVA results for the behavioral variants that did not differ significantly across communities. PL-East = Punta Laguna – East community; PL-West = Punta Laguna – West community; *Not significant as critical value is 0.013 when Bonferroni's correction was applied (see Methods); †Not significant as critical value is 0.017 when Bonferroni's correction was applied (see Methods). (TIF) [file pone.0024400.s004.tif]
